# Supplementary material for: Double NPY motifs at the N-terminus of the yeast t-SNARE Sso2 synergistically bind Sec3 to promote membrane fusion
Source: eLife. 2022 Aug 18;11:e82041. doi: 10.7554/eLife.82041 (PMC9427108; doi:10.7554/eLife.82041)
Supplement: Supplementary file 1. [file elife-82041-supp1.docx]

**Supplementary file 1. Bacteria strains**

| SFNB1223 | pRS416-TPIpro-GFP-Snc1 | Lab collection |
| --- | --- | --- |
| NRB1303 | pRS306-Sec3-3xGFP | Lab collection |
| NRB1312 | pRS306-Sec4-GFP | Lab collection |
| NRB1644 | pRS305-Sec3-His-3xFlag (integration into SEC3 locus) | Lab collection |
| NRB1652 | hemizap-pRS306-SSO2pro-*SSO2* | This study |
| NRB1653 | hemizap-pRS306-SSO2pro-*sso2*-E8A *(sso2M1)* | This study |
| NRB1654 | hemizap-pRS306-SSO2pro-*sso2*-Y7A (*sso2M2*) | This study |
| NRB1655 | hemizap-pRS306-SSO2pro-*sso2*-N5AP6A (*sso2M3*) | This study |
| NRB1656 | hemizap-pRS306-SSO2pro-*sso2*-N5AP6AY7A (*sso2M4*) | This study |
| NRB1657 | hemizap-pRS306-SSO2pro-*sso2-*N5AP6AY7AE8A (*sso2M5*) | This study |
| NRB1658 | hemizap-pRS306-SSO2pro-*sso2-*N11AP12AY13A (*sso2M6*) | This study |
| NRB1659 | hemizap-pRS306-SSO2pro-*sso2-*N5AP6AY7AE8A-N11AP12AY13A (*sso2M7*) | This study |
