# Supplementary material for: Double NPY motifs at the N-terminus of the yeast t-SNARE Sso2 synergistically bind Sec3 to promote membrane fusion
Source: eLife. 2022 Aug 18;11:e82041. doi: 10.7554/eLife.82041 (PMC9427108; doi:10.7554/eLife.82041)
Supplement: Supplementary file 2. [file elife-82041-supp2.docx]

**Supplementary file 2. Yeast strains**

| NY3305 | *Mat-a his3∆1 lys2-∆0 leu2∆0 ura3∆0 sso1∆::KanMX4 SSO2* | This study |
| --- | --- | --- |
| NY3306 | *Mat-a his3∆1 lys2-∆0 leu2∆0 ura3∆0 sso1∆::KanMX4 sso2M1* | This study |
| NY3307 | *Mat-a his3∆1 lys2-∆0 leu2∆0 ura3∆0 sso1∆::KanMX4 sso2M2* | This study |
| NY3308 | *Mat-a his3∆1 lys2-∆0 leu2∆0 ura3∆0 sso1∆::KanMX4 sso2M3* | This study |
| NY3309 | *Mat-a his3∆1 lys2-∆0 leu2∆0 ura3∆0 sso1∆::KanMX4 sso2M4* | This study |
| NY3310 | *Mat-a his3∆1 lys2-∆0 leu2∆0 ura3∆0 sso1∆::KanMX4 sso2M5* | This study |
| NY3311 | *Mat-a his3∆1 lys2-∆0 leu2∆0 ura3∆0 sso1∆::KanMX4 sso2M6* | This study |
| NY3312 | *Mat-α his3∆1 lys2-∆0 leu2∆0 ura3∆0 sso1∆::KanMX4 sso2M7* | This study |
| NY3313 | *Mat-a his3∆1 lys2-∆0 leu2∆0 ura3∆0 sso1∆::KanMX4 SSO2 [GFP-SNC1::URA3 CEN6]* | This study |
| NY3314 | *Mat-a his3∆1 lys2-∆0 leu2∆0 ura3∆0 sso1∆::KanMX4 sso2M5 [GFP-SNC1::URA3 CEN6]* | This study |
| NY3315 | *Mat-a his3∆1 lys2-∆0 leu2∆0 ura3∆0 sso1∆::KanMX4 sso-M6 [GFP-SNC1::URA3 CEN6]* | This study |
| NY3316 | *Mat-α his3∆1 lys2-∆0 leu2∆0 ura3∆0 sso1∆::KanMX4 sso2M7 [GFP-SNC1::URA3 CEN6]* | This study |
| NY3317 | *Mat-a his3∆1 lys2-∆0 leu2∆0 ura3∆0 sso1∆::KanMX4 SSO2[pRS306-SEC4-GFP::URA]* | This study |
| NY3318 | *Mat-a his3∆1 lys2-∆0 leu2∆0 ura3∆0 sso1∆::KanMX4 sso2M5 [pRS306-SEC4-GFP::URA]* | This study |
| NY3319 | *Mat-α his3∆1 lys2-∆0 leu2∆0 ura3∆0 sso1∆::KanMX4 sso2M7 [pRS306-SEC4-GFP::URA]* | This study |
| NY3320 | Mat-a his3∆1 lys2-∆0 leu2∆0 ura3∆0 sso1∆::KanMX4 *SSO2* [pRS306-Sec3-3xGFP::URA3] | This study |
| NY3321 | Mat-a his3∆1 lys2-∆0 leu2∆0 ura3∆0 sso1∆::KanMX4 *sso2M5* [pRS306-Sec3-3xGFP::URA3] | This study |
| NY3322 | Mat-α his3∆1 lys2-∆0 leu2∆0 ura3∆0 sso1∆::KanMX4 *sso2M7* [pRS306-Sec3-3xGFP::URA3] | This study |
| HY3447 | *Mat-a his3∆1 lys2-∆0 leu2∆0 ura3∆0 sso1∆::KanMX4 SSO2* [pRS305-Sec3-His-3xFlag | This Study |
| HY3448 | *Mat-α his3∆1 lys2-∆0 leu2∆0 ura3∆0 sso1∆::KanMX4 sso2M7*  [pRS305-Sec3-His-3xFlag] | This study |
