# Supplementary figures and images for: Double NPY motifs at the N-terminus of the yeast t-SNARE Sso2 synergistically bind Sec3 to promote membrane fusion
Source: eLife. 2022 Aug 18;11:e82041. doi: 10.7554/eLife.82041 (PMC9427108; doi:10.7554/eLife.82041)

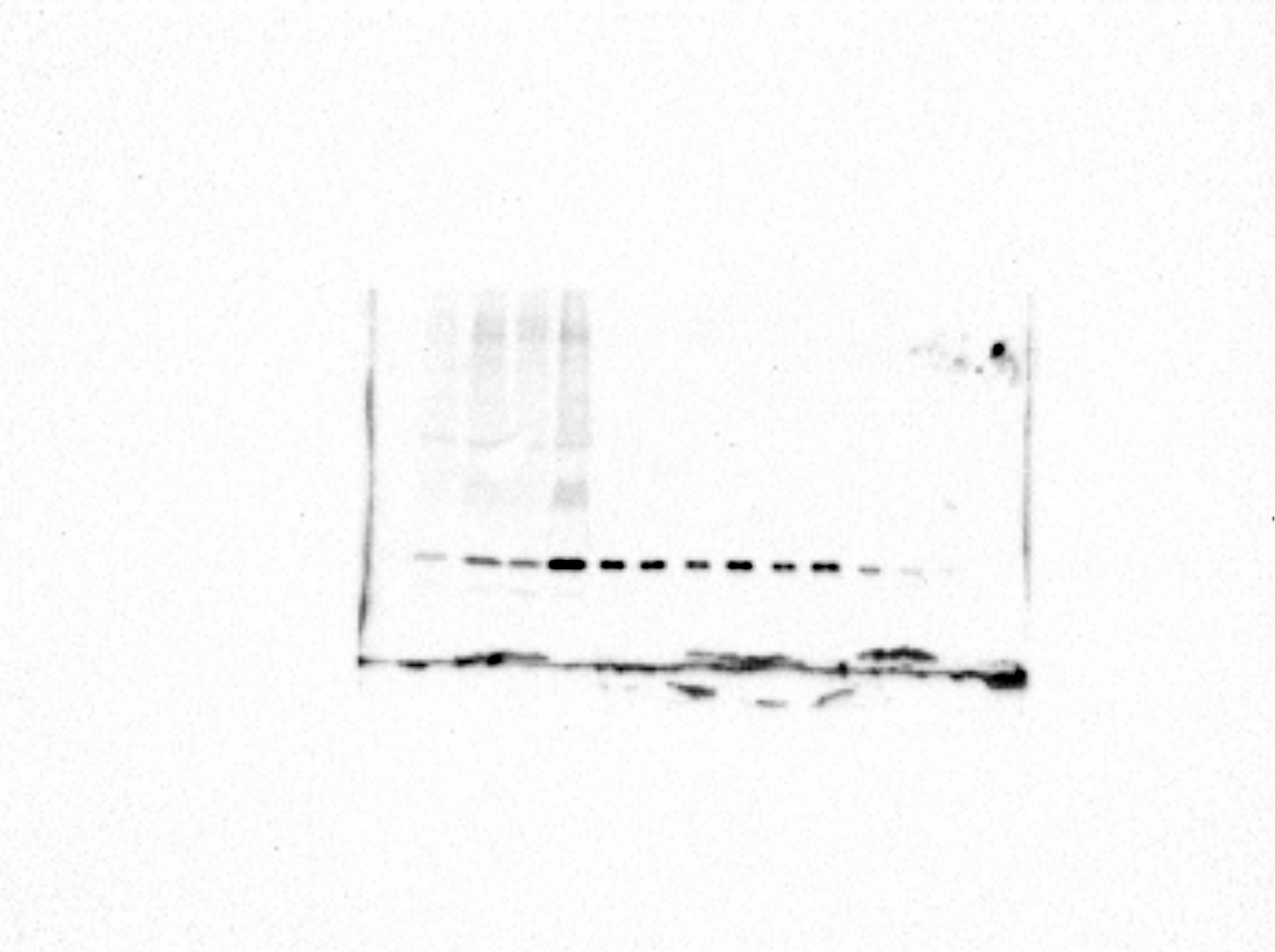

Supplement: Source data 1. [file elife-82041-data1.zip › Source data 1/Figure 4 C.tif]

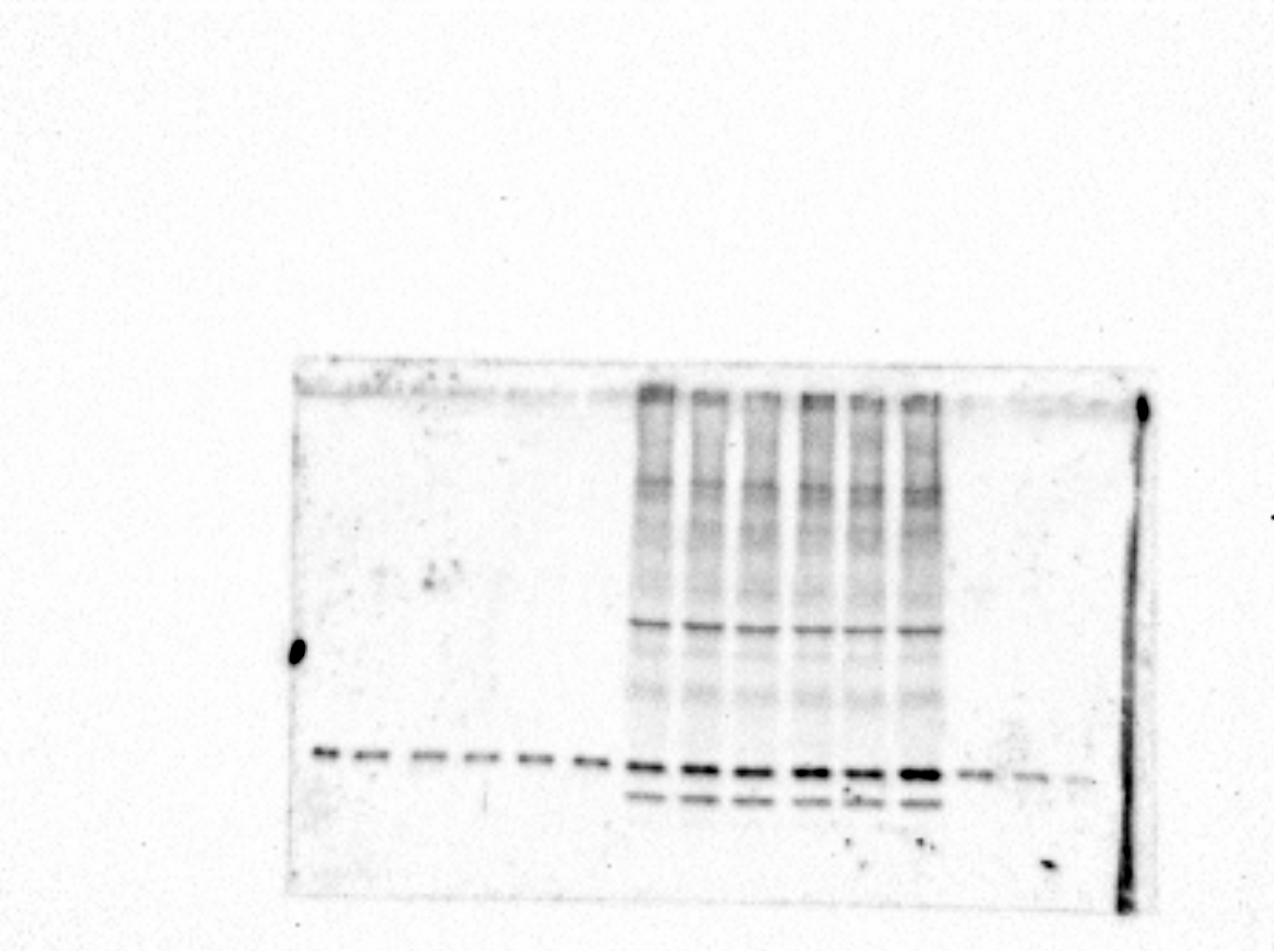

Supplement: Source data 1. [file elife-82041-data1.zip › Source data 1/Figure 4-figure supplement 1 C.tif]

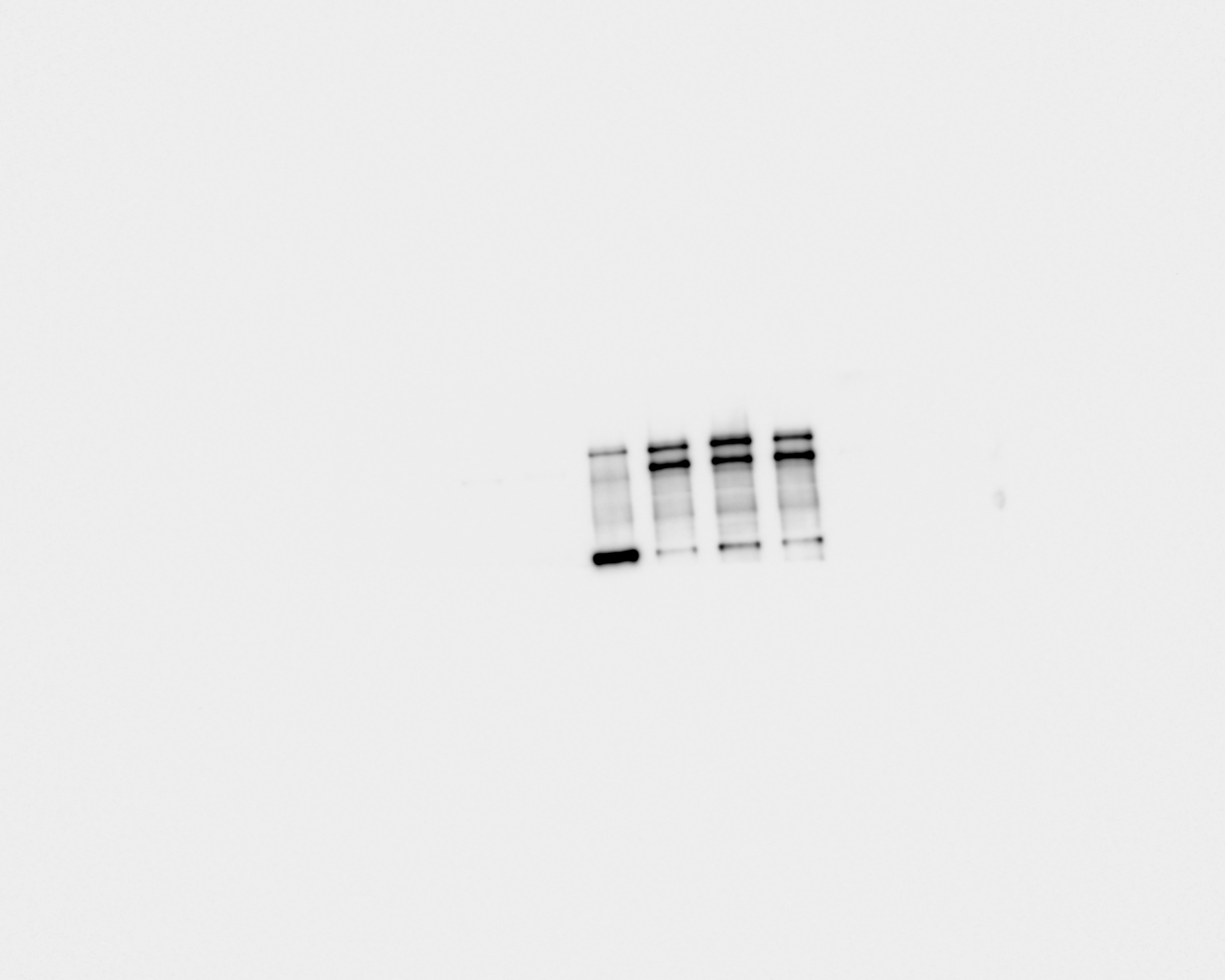

Supplement: Source data 1. [file elife-82041-data1.zip › Source data 1/Figure 8-figure supplement 2_Sec3.tif]

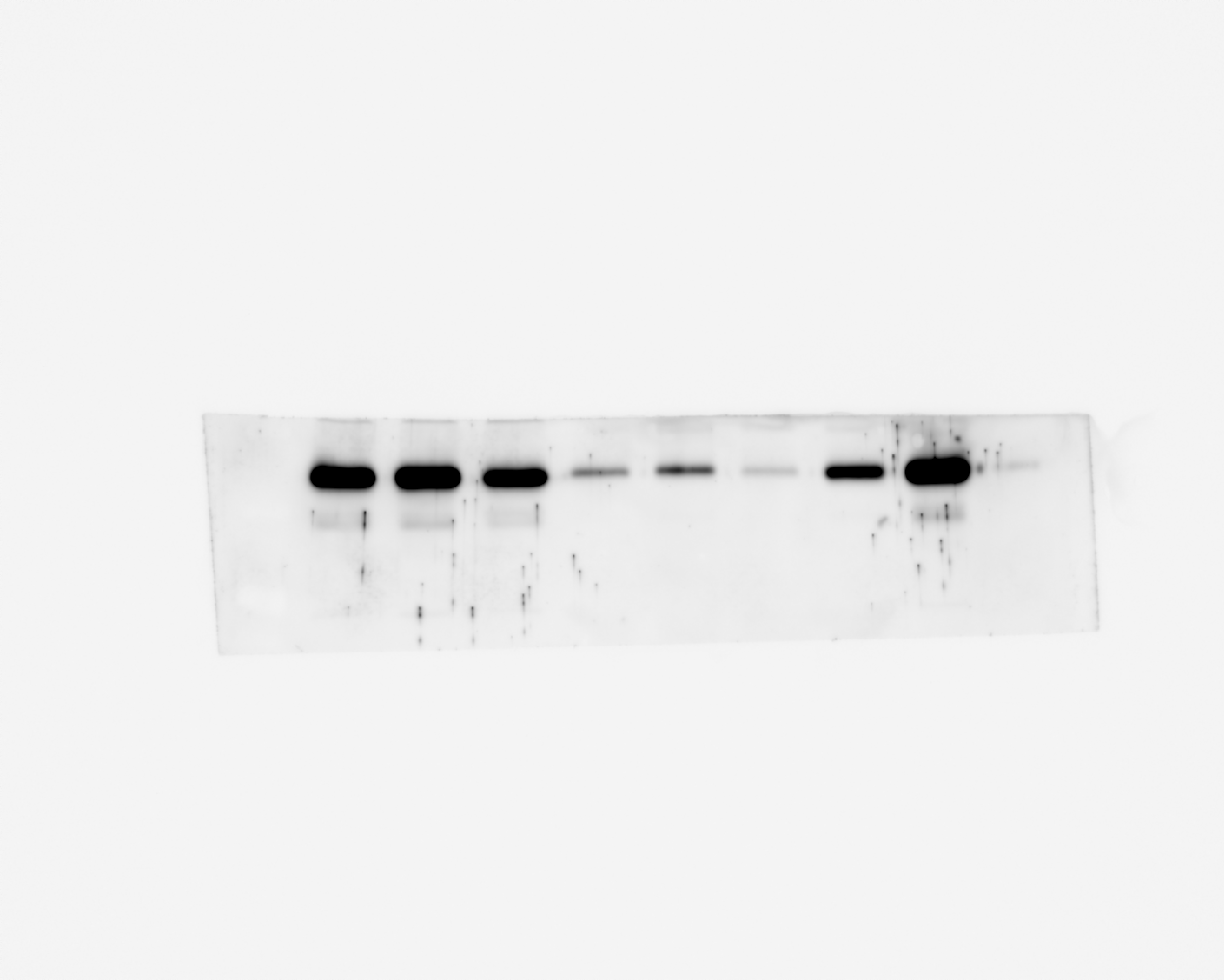

Supplement: Source data 1. [file elife-82041-data1.zip › Source data 1/Figure 8-figure supplement 2_Sso2.tif]

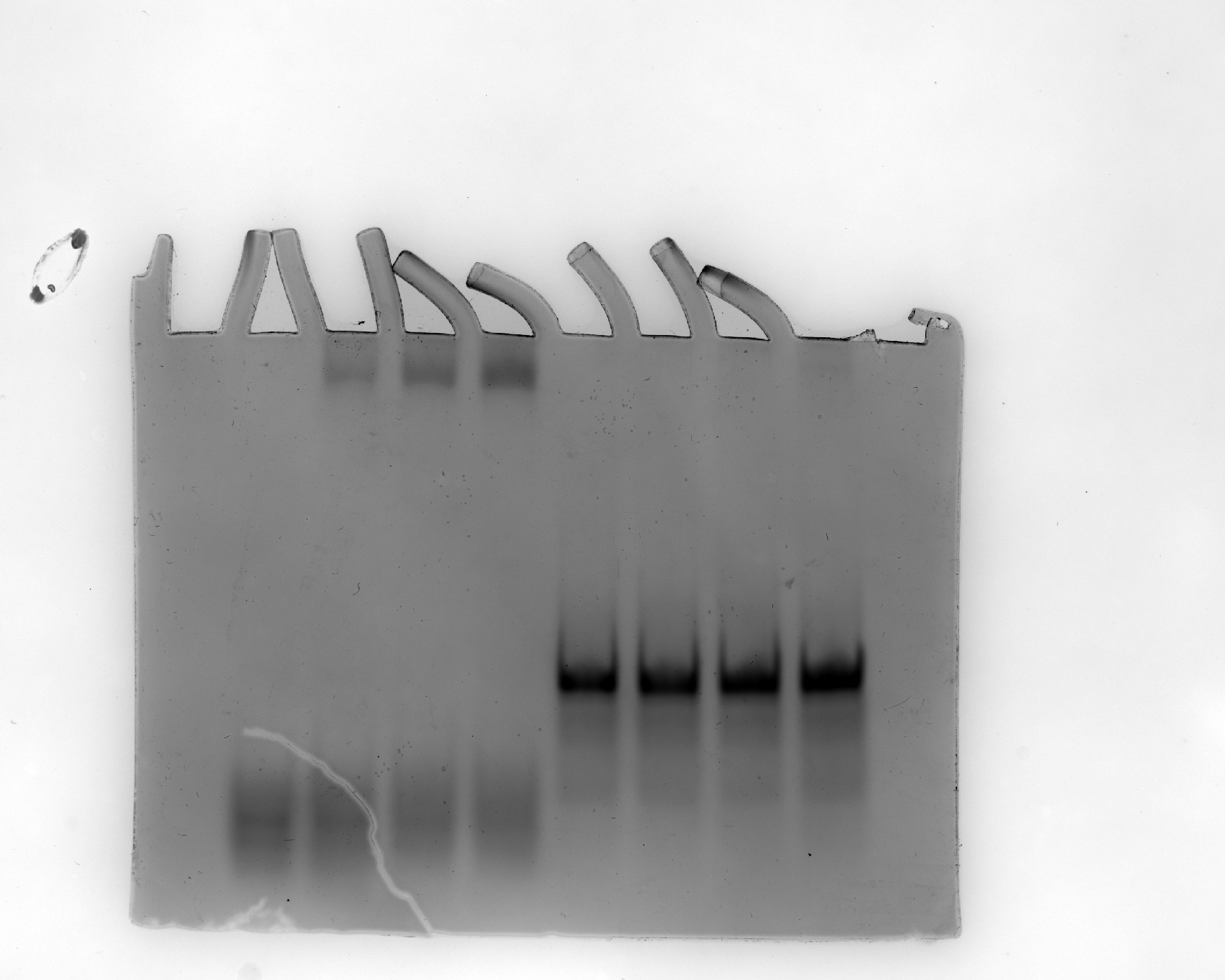

Supplement: Source data 1. [file elife-82041-data1.zip › Source data 1/Figure 8-figure supplment 1 A.jpg]

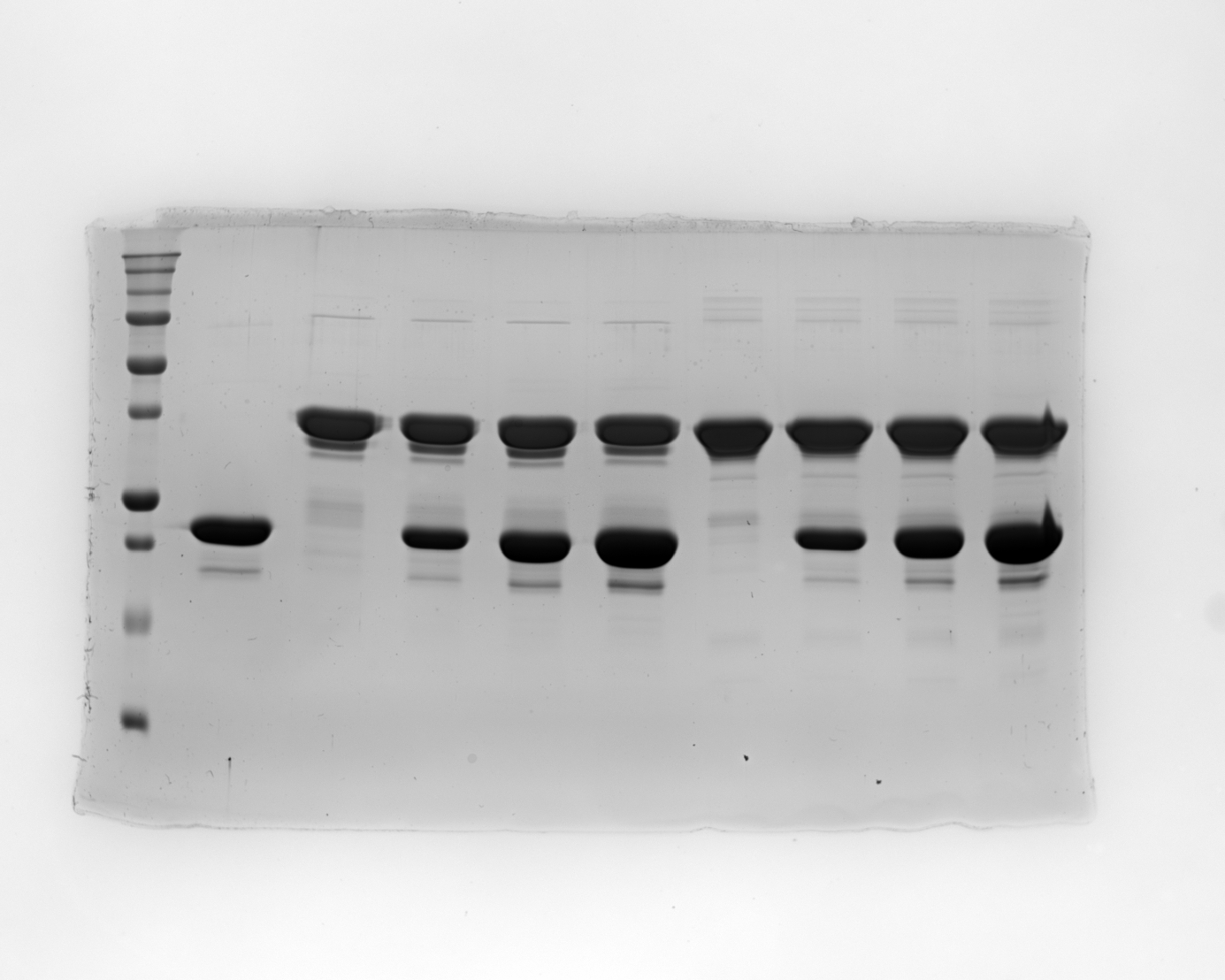

Supplement: Source data 1. [file elife-82041-data1.zip › Source data 1/Figure 8-figure supplment 1 B.jpg]

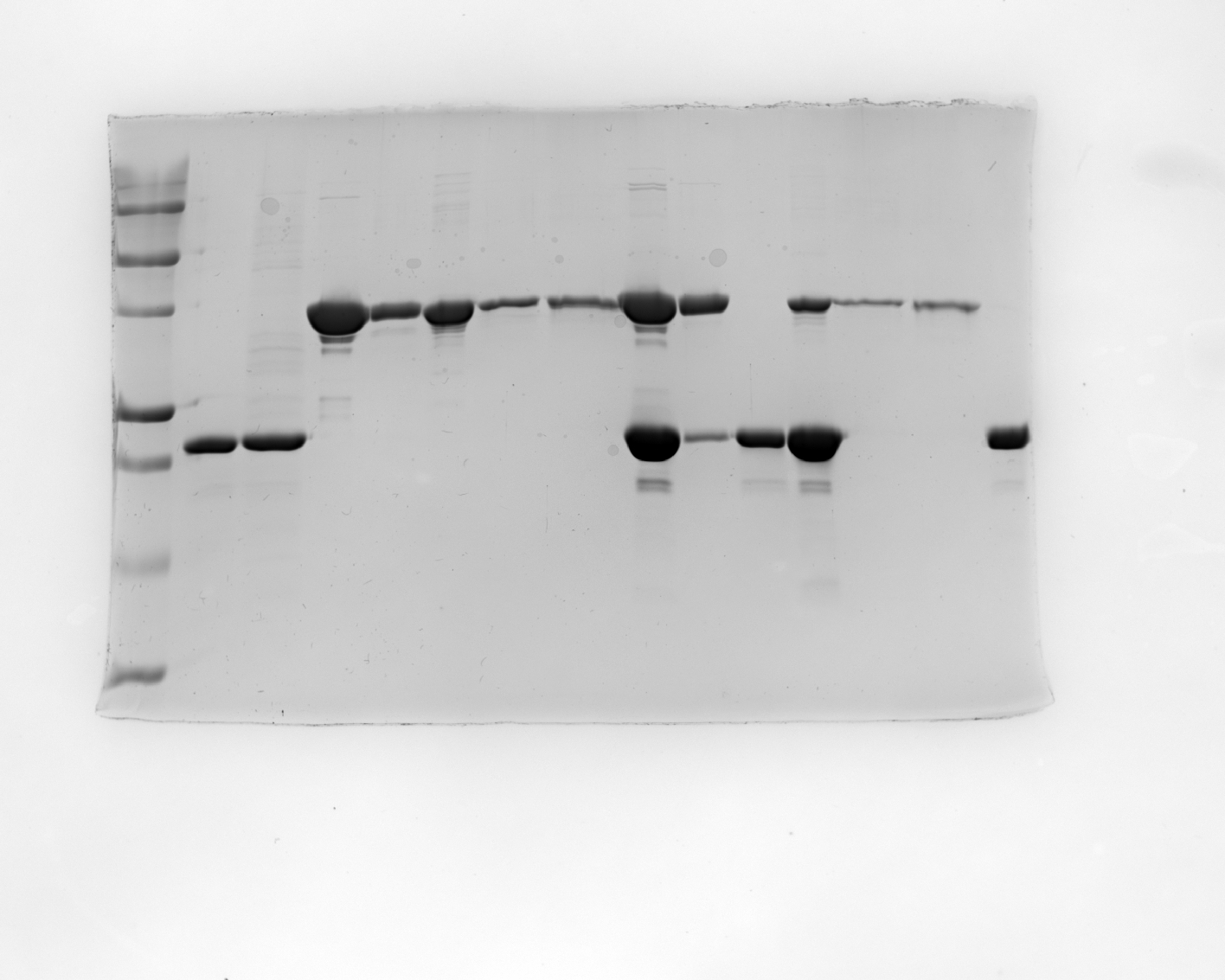

Supplement: Source data 1. [file elife-82041-data1.zip › Source data 1/Figure 8-figure supplment 1 D1.jpg]

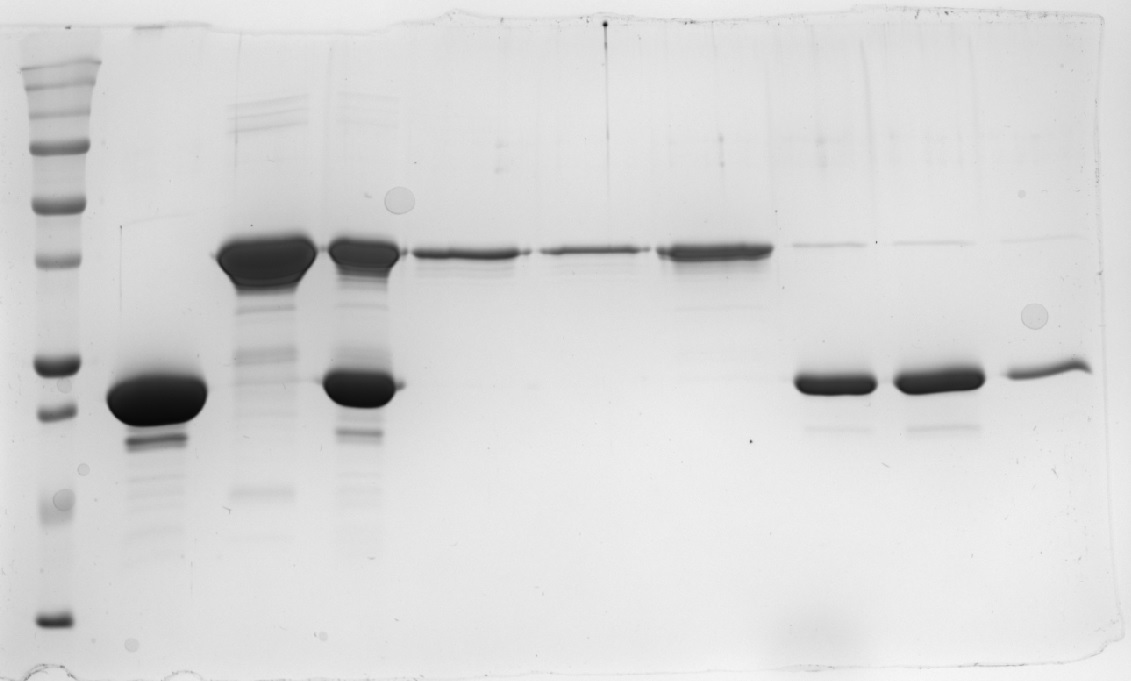

Supplement: Source data 1. [file elife-82041-data1.zip › Source data 1/Figure 8-figure supplment 1 D2.jpg]
